# Bronchial thermoplasty in severe asthma: a real-world study on efficacy and gene profiling

**DOI:** 10.1186/s13223-022-00680-4

**Published:** 2022-05-09

**Authors:** Nicola Facciolongo, Martina Bonacini, Carla Galeone, Patrizia Ruggiero, Francesco Menzella, Giulia Ghidoni, Roberto Piro, Chiara Scelfo, Chiara Catellani, Alessandro Zerbini, Stefania Croci

**Affiliations:** 1 Pneumology Unit, Azienda Unità Sanitaria Locale-IRCCS Di Reggio Emilia, Reggio Emilia, Italy; 2 Unit of Clinical Immunology, Allergy and Advanced Biotechnologies, Azienda Unità Sanitaria Locale-IRCCS Di Reggio Emilia, Reggio Emilia, Italy; 3 Department of Medical Specialties, Pneumology Unit, Arcispedale Santa Maria Nuova, Azienda Unità Sanitaria Locale-IRCCS, 42123 Reggio Emilia, Italy

**Keywords:** Bronchial thermoplasty, Severe asthma, Gene expression, Real-time PCR, Smooth muscle, Bronchial biopsies

## Abstract

**Background:**

Bronchial thermoplasty (BT) is an effective treatment in severe asthma. How to select patients who more likely benefit from BT is an unmet clinical need. Moreover, mechanisms of BT efficacy are still largely unknown. We sought to determine BT efficacy and to identify potential mechanisms of response.

**Methods:**

This retrospective cohort study evaluated clinical outcomes in 27 patients with severe asthma: 13 with T2-high and 14 with T2-low endotype. Expression levels of 20 genes were compared by real-time PCR in bronchial biopsies performed at the third BT session versus baseline. Clinical response was measured based on Asthma Control Questionnaire (ACQ) score < 1.5, asthma exacerbations < 2, oral corticosteroids reduction of at least 50% at 12 months post-BT. Patients were classified as responders when they had at least 2 of 3 outcome measures.

**Results:**

81% of patients were defined as responders. BT induced a reduction in alpha smooth muscle actin (ACTA2) and an increase in CD68, fibroblast activation protein-alpha (FAP), alpha-1 and alpha-2 type I collagen (COL1A1, COL1A2) gene expression in the majority of patients. A higher reduction in ubiquitin carboxy-terminal-hydrolase L1 (PGP9.5) mRNA correlated with a better response based on Asthma Quality of Life Questionnaire (AQLQ). Lower changes in CD68 and FAP mRNAs correlated with a better response based on ACQ. Lower levels of occludin (OCLN), CD68, connective tissue growth factor (CTGF), higher levels of secretory leukocyte protease inhibitor (SLPI) and lower changes in CD68 and CTGF mRNAs were observed in patients who had less than 2 exacerbations post-BT. Lower levels of COL1A2 at baseline were observed in patients who had ACQ < 1.5 at 12 months post-BT.

**Conclusions:**

BT is effective irrespective of the asthma endotypes and seems associated with airway remodelling. Quantification of OCLN, CD68, CTGF, SLPI, COL1A2 mRNAs could be useful to identify patients with better results.

*Trial registration*: The study protocol was approved by the Local Ethics Committee (Azienda USL-IRCCS of Reggio Emilia—Comitato Etico Area Vasta Nord of Emilia Romagna; protocol number: 2019/0014076) and all the patients provided written informed consent before participating in the study.

**Supplementary Information:**

The online version contains supplementary material available at 10.1186/s13223-022-00680-4.

## Background

The heterogeneous and inflammatory background of severe asthma is today richly documented [1]. High-throughput analyses contributed to better define the current and well known multiple endotypes and phenotypes of severe refractory asthma (SRA) [2]. However, it is still debated how to choose the best therapeutic options for the right asthma phenotype. Immunologic profile includes two severe asthma endotypes: T helper type 2 cell high (T2-high), associated to serum biomarkers as eosinophils and immunoglobulin E (IgE), and T helper type 2 cell low (T2-low) characterized by neutrophilic or paucigranulocytic inflammatory pattern and airway damage associated with mucus gland hyperplasia and hyper secretion, airway hyperreactivity, remodelling, and corticosteroid insensitivity [3].

Bronchial thermoplasty (BT) is considered an effective treatment in the T2-low endotype [4], however it could be a therapeutic option for all patients whose symptoms are not controlled with standard of care and despite monoclonal antibody treatments [5]. BT is an endoscopic procedure based on the local delivery of radio frequency at 65 °C to the airways with a device called Alair™ Catheter (Boston Scientific, Natick, MA, USA) [6]. Several clinical trials have demonstrated BT effectiveness especially on clinical outcomes, particularly in the reduction of exacerbations [7–9]. Long-term effects of BT have been documented by observational studies [10].

However, the discussion concerning effectiveness of BT and its mechanism of action remains open [11]. These include changes in function and structure of airway smooth muscles, nerve fibers, inflammatory cells/mediators and components of the extracellular matrix [12–17]. Furthermore, a key aspect yet to be clarified is the identification of potential predictive response factors [18], to identify those patients who more likely benefit from BT. An operator-dependent variable that can influence response may be the number of activations delivered during BT sessions, which appears to play an important role in determining favorable clinical outcomes [19].

The aims of this study were to determine efficacy of BT, to identify genes modulated by BT and associations between gene expression levels in bronchial biopsy specimens and clinical outcomes. We investigated the expression of 20 genes, selected following a hypothesis-driven approach starting from and expanding literature data [6, 13–17, 20–24]. In particular, we analyzed expression of genes reflecting airway remodelling: alpha smooth muscle actin (ACTA2) marker of airway smooth muscle cells, cadherin 1 (CDH1) marker of epithelial cells, connective tissue growth factor (CTGF), fibroblast activation protein alpha (FAP), alpha-1 and alpha-2 chain type I collagen (COL1A1, COL1A2), galectin-3 (LGAL3) markers of stroma. We investigated expression of genes mirroring airway inflammation: CD45, CD68, interleukin (IL)-4, IL-5, IL-6, IL-13, IL-17. We studied expression of innervation markers: ubiquitin carboxy-terminal hydrolase L1 (UCHL1/PGP9.5) and occludin (OCLN) as well as genes reported to be associated with asthma: periostin (POSTN), galectin-3 (LGAL3), secretory leukocyte protease inhibitor (SLPI), IL-4, IL-5, IL-13, IL-17. We investigated genes coding high temperature receptors (transient receptor potential cation channel subfamily V member 1 and member 2: TRPV1 and TRPV2) [22, 23] because BT is based on heat delivery. Finally we analyzed the expression of SLPI which has emerged as the most promising potential marker of response to BT in the work by Ano S et al. [24].

## Methods

### Study population

A total of 27 adult patients who underwent BT at Pulmonology Unit of the Azienda USL-IRCCS of Reggio Emilia were enrolled. Patients were affected by severe refractory asthma according to ERS/ATS classification [25].

The study protocol was approved by the Local Ethics Committee (Azienda USL-IRCCS of Reggio Emilia—Comitato Etico Area Vasta Nord of Emilia Romagna; protocol number: 2019/0014076) and all the patients provided written informed consent before participating in the study.

Clinical data, blood biomarkers, asthma medications, patient reported outcomes in terms of asthma quality of life questionnaire (AQLQ) and asthma control questionnaire (ACQ) were collected. Data were assessed at baseline with a run-in period of 12 months (before BT, T0), at 2 months during the last BT session (T2) and 12 months after the last BT session (T12). Patients were referred by pulmonologists from Azienda USL-IRCCS of Reggio Emilia and other Italian pulmonology Units. The data was then collected in a single dataset. On the basis of the cut-off for anti-IL-5 therapy (blood eosinophils count ≥ 300 cells/µl), patients were classified in the T2-high and T2-low endotypes. The first was defined as eosinophilic (blood eosinophils count ≥ 300 cells/µl), eosinophilic and allergic (blood eosinophils count ≥ 300 cells/µl, IgE ≥ 76 kU/l, perennial inhalant allergens), allergic (IgE ≥ 76 kU/l and perennial inhalant allergens). The T2-low endotype was defined by neutrophilic or paucigranulocytic inflammation, blood eosinophil count < 300 cells/ µl and absence of atopy.

### Measures to determine BT response

Outcome measures included: (1) ACQ score < 1.5 at T12; (2) < 2 asthma exacerbations at T12 versus T2; (3) oral corticosteroids (OCSs) reduction of at least 50% at T12 versus baseline. Patients were classified as responders when they had at least 2 of the 3 outcome measures. Patients who did not receive OCSs at baseline were classified as responders when they had one of the other outcome measures.

### BT procedure

BT was performed with the device called Alair™ Catheter (Boston Scientific, Natick, MA, USA). All patients underwent 3 sessions of BT which consisted in the treatment of right lower lobe (first session), left lower lobe (second session) and right and left upper lobes (third session). Sessions were performed every four weeks. No heat activations were released in the middle lobe [13].

### Airway sample collection

Bronchial biopsy specimens were performed in the left lower lobe at baseline (as T0) and in the right lower lobe after two months during the third BT session (T2) which was administered in the upper lobes. Bronchial biopsies were collected before heat delivery. At least two samples were embedded in Tissue Tek II OCT (Miles Scientific, Naperville, IL, USA), frozen within 15 min at − 20 °C, and finally stored at − 80 °C.

### RNA extraction and cDNA preparation

Biopsies were recovered from thawed OCT and RNA was extracted with the RNA/DNA/Protein Purification Plus Kit (Norgen Biotek). Tissue homogenization was made effective by the use of pestles. NanoDrop instrument (Thermo Scientific) was used for RNA quantification, then 62.5 ng of RNA were reverse transcribed with the PrimeScrip RT Reagent Kit with gDNA Eraser (Takara) according to the manufacturer's instructions. cDNA was diluted 1:2 with DNase and RNase-free water and stored at − 20° C.

### Real-time PCR

Real-time PCR was performed with the SYBR Premix Ex Taq II (Tli RNaseH Plus) containing the ROX Reference Dye (Takara) and QuantiTect primer assays (Qiagen). Catalog numbers of the primers are listed in Supplementary Table 1. cDNA (1 μL) was amplified in 25 μL PCR mix. Amplification products were checked by dissociation curve analysis. Glyceraldehyde 3-phosphate dehydrogenase (GAPDH) was used as housekeeper gene for data normalization. Real-time PCR results are shown as normalized expression: 2-ΔCt (ΔCt = Ct mRNA target – Ct GAPDH) so that they might be comparable among different laboratories. Amplification was carried out with the 7300 Applied Biosystem instrument (Thermofisher).

### Statistical analysis

Statistical analysis was performed with GraphPad Prism 6 software using non-parametric tests. For comparison between two groups, Wilcoxon test was applied for paired data, while Mann–Whitney U test was applied for unpaired data. For comparison between three groups, Kruskal–Wallis test with Dunns correction was used. Spearman test was used to determine correlations between two variables. Categorical values were compared with Fisher exact test. P values < 0.05 were considered statistically significant.

## Results

### Demographic results at baseline

A total of 27 patients with severe refractory asthma according to ERS/ATS 2014 guidelines and GINA 2018 classification underwent BT from 2012 to 2018. Demographic, clinical features and comorbidities of the cohort of patients are shown in Table 1.

**Table 1:** Demographic and clinical characteristics of the patients

Demographic and Clinical Characteristics	T0
No. of patients	27
Age y, mean ± SD	52 ± 10
Female, n (%)	14 (52)
Age onset y, mean ± SD	36 ± 12
Ex smokers, n (%)	5 (18)
Atopics, n (%)	6 (22)
Switch from Omalizumab, n (%)	5 (18)
Phenotypes	
T2-high, n (%)	13 (48)
* Eosinophilic, n (%)*	7 (26)
* Eosinophilic and Allergic, n (%)*	3 (11)
* Only Allergic, n (%)*	3 (11)
T2-low, n (%)	14 (52)
* Neutrophilic, n (%)*	14 (52)
Comorbidities	
Gastroesophageal reflux disease (GERD), n (%)	14 (52)
Chronic rhinosinusitis with nasal polyps (CRSwNP), n (%)	9 (33)
BT Activations mean (SD)	193 ± 39

*GERD* gastro-esophageal reflux disease, *CRSwNP* chronic rhinosinusitis with nasal polyposis

### BT clinical outcomes

According to outcome measures, 81% of the patients were defined as responders to BT treatment (Table 2). In particular, 77% of the patients with the T2-high endotype and 86% with the T2-low endotype were responders.

**Table 2:** Clinical criteria for definition of patients with a better response to BT

T2-high endotype				T2-low endotype			
Patient ID	OCS reduction at T12 versus baseline (%)	Number of exacerbations at T12 versus T2	ACQ score at T12	Patient ID	OCS reduction at T12 versus baseline (%)	Number of exacerbations at T12 versus T2	ACQ score at T12
1	100	1	2.0	2	50	1	2.5
3	100	0	1.5	5	80	2	2.0
4	0	2	2.4	9	100	1	1.0
6	50	1	2.6	11	100	0	2.6
7	100	0	0	13	100	1	1.6
8	100	1	0.6	14	50	0	1.4
10	50	2	1.2	15	0	2	1.0
12	100	0	1.6	18	100	1	2.0
16	75	1	1.2	19	NA	0	1.2
17	75	2	1.4	20	100	1	1.8
25	50	2	1.4	21	75	1	1.2
26	100	2	2.1	22	NA	1	1.6
27	50	3	2.5	23	50	1	1.6
				24	100	2	1.2
Total responder: 22 (81%)							

Patients were classified as responders when they had at least 2 of the 3 outcome measures: (1) ACQ score < 1.5 at T12; (2) < 2 asthma exacerbations at T12 versus T2; (3) OCSs reduction of at least 50% at T12 versus baseline. Patients who did not receive OCSs at baseline (NA) were classified as responders when they had one of the other outcome measures

*OCS* oral corticosteroids, *NA* (not applicable) patients who did not take OCS at baseline, Colored patient boxes = responders

Clinical and airway functional parameters before and 12 months after BT are described in Table 3. Significant improvements in patients reported outcomes (ACQ and AQLQ scores), rate of exacerbations and airway functional data were observed (Table 3). The overall consumption of OCSs scaled down from 20 to 5 mg daily after 1 year (p < 0.0001). At baseline, 89% of patients received OCSs and among these, 40% stopped OCSs at T12 and 55% reduced OCS daily dose by 50% (p < 0.0058). Among patients taking OCS, 7 had blood eosinophil counts ≥ 300 cells / µl (535.71 ± 241.51 cells / µl) with a mean prednisone dose of 16.4 ± 8.4 mg, while 10 patients had blood eosinophils ≥ 150 cells / µl (452.0 ± 238.9 cells / ml) with a mean prednisone dose of 17.7 ± 7.9 mg.

**Table 3:** Clinical characteristics of the patients before and after BT

Parameter	T0	T12	*p*-value
No. of patients	27	27	_
Total serum IgE KU/l, median [IQR]	65 [30–160]	87 [27–272]	0.2552
Blood eosinophil count cells/µl, median [IQR]	140 [20–410]	340 [120–640]	0.1533
Lung function, pre-bronchodilator			
FEV_1_%,median [IQR]	71 [63–94]	67 [59–87]	0.0179
FEV_1_ l, median [IQR]	2.43 [1.86–3.22]	1.97 [1.5–2.68]	0.0103
FVC %, median [IQR]	99 [89–114]	86 [77–102]	0.0011
FVC l, median [IQR]	3.62 [2.90–4.62]	3.19 [2.57–3.89]	0.0051
Asthma concomitant medication			
Oral corticosteroid-prednisone or the equivalent mg, median [IQR]	20 [5–25]	5 [0–12.5]	< 0.0001
Patients taking OCS, n (%)	24 (89)	15 (55)	0.0058
Patients taking LAMA, n (%)	12 (44)	21 (77)	0.0244
Patients taking LTRA, n (%)	10 (37)	6 (22)	1
Patients taking theophylline, n (%)	4 (15)	0 (0)	0.1110
Assigned adapted GINA* step			
Step 4, n (%)	2 (7)	3 (11)	1
Step 5, n (%)	25 (93)	24 (89)	1
Asthma control			
Exacerbations/y, median [IQR]	6 [3–10]	2 [1–3]	< 0.0001
ED visits/hospitalizations/y, median [IQR]	0 [0–1]	0 [0–0]	0.0022
Patient reported outcomes			
AQLQ score, median [IQR]	2.6 [2.1–3.0]	5.5 [4.4–6.0]	< 0.0001
ACQ score, median [IQR]	3.8 [3.2–4.6]	1.6 [1.2–2.0]	< 0.0001

^*^GINA 2019 asthma guidelines

Data were compared with Mann Withney U test (continuous variables) and Fisher exact test (categorical variables)

P < 0.05 were considered statistically significant. *IQR* interquartile range

### Characterization of bronchial gene expression at baseline

IL-4, IL-13 and IL-17 mRNAs were not detected in bronchial biopsies at baseline. IL-5 was detected in 9/27 samples while TRPV2 was detected in 21/27 samples with low expression (median Ct of IL-5: 34.2, IQR: 33.4–34.4; median Ct of TRPV2: 32.9, IQR: 31.3–33.9). The other investigated transcripts were expressed in all the biopsies, showing variable expression levels among different patients. Coefficient of variations (CVs) ranged from 42.1% to 168.0% (Supplementary Table 2). The genes that showed more variability (CV > 100%) were FAP and IL-6, while those which showed less variability (CV < 50%) were CD68, LGAL3, CDH1.

Since patients were receiving OCSs, we determined if gene expression levels were associated with the dose of OCSs. Patients were divided in three groups based on OCS dose: ≤ 5 mg; 5–25 mg; ≥ 25 mg. OCSs did not affect the expression of the investigated genes (Additional file 2: Figure S1).

No differences were observed in gene expression levels when patients were stratified in endotypes (data not shown).

### Effects of BT treatment on gene expression

**Fig. 1:**
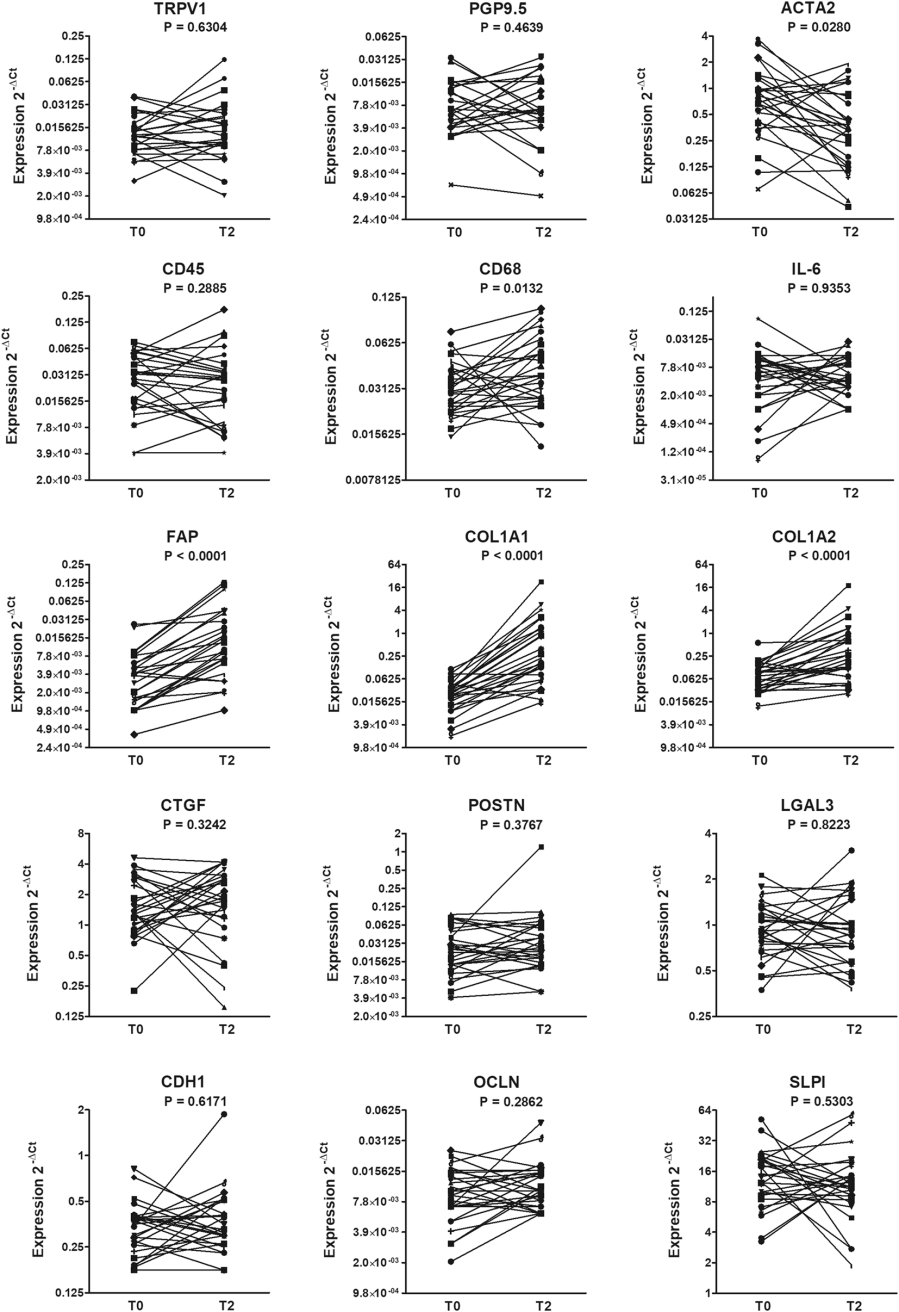
Changes in gene expression following BT treatment. Gene expression levels in bronchial biopsies from each patient (n = 27) at baseline (T0) and at the third BT session (T2) were determined by real-time PCR. Gene expressions were calculated by the 2^− ΔCt^ method using the GAPDH as housekeeper gene. Data were analyzed by Wilcoxon test. Y axis is in logarithmic scale

To determine the effects of BT treatment, gene expression levels were compared in bronchial biopsies performed during the last BT session (T2) *versus* baseline. Patients showed to be heterogeneous regarding changes in gene expression induced by BT treatment (Fig. 1). Overall, ACTA2 showed a significant reduction and CD68, FAP, COL1A1 and COL1A2 showed a significant increase at T2 *versus* T0 (P value < 0.05, Fig. 1). No significant changes were observed in the other markers analyzed. Supplementary Table 3 reports the fraction of patients who had changes greater or equal to two-fold. Specifically, 52% of patients showed at least a two-fold decrease in ACTA2 while 70%, 89%, 63% of patients showed at least a two-fold increase in FAP, COL1A1, COL1A2 respectively. Changes in CD68 gene expression were mainly lower than two-fold, although statistically significant.

### Correlations between changes in gene expression induced by BT treatment and differences in patient reported outcomes

**Fig. 2:**
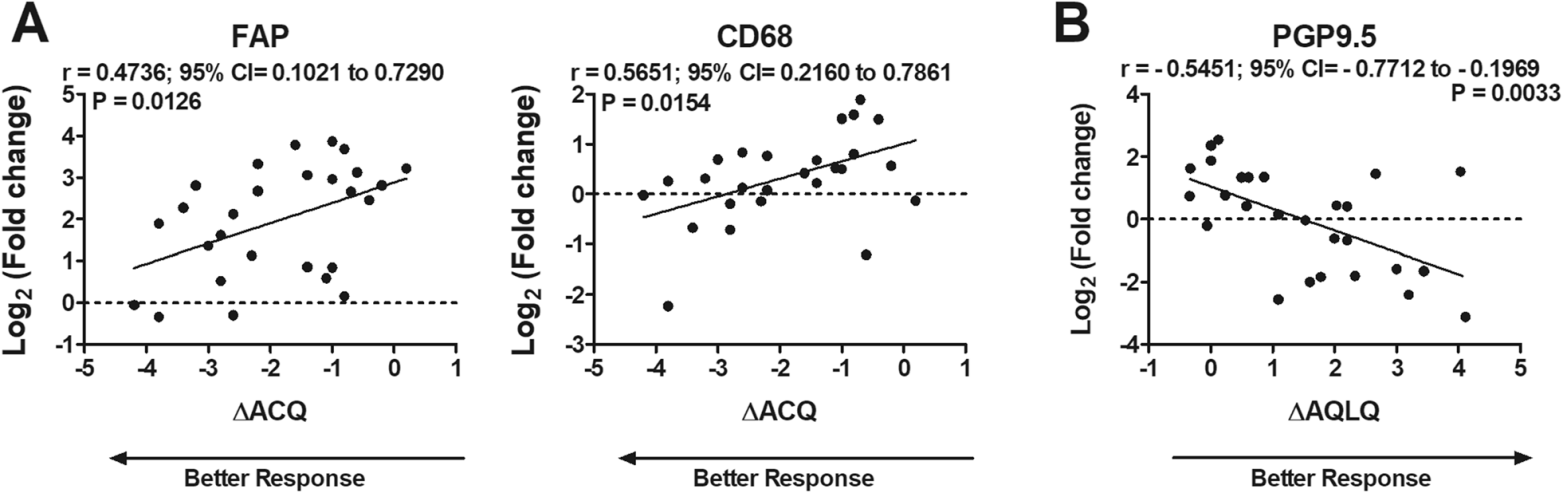
Changes in gene expression correlated with patients reported outcomes during BT treatment. Dot plot visualization of correlations between fold changes in gene expression and differences in ACQ (**A**) and AQLQ (**B**) scores between T2 and T0. Fold changes in gene expression were determined by real-time PCR relative to gene expression at T0 through the 2^−ΔΔCt^ method. Data were analyzed by Spearman's correlation test (n = 27). r = correlation coefficient; 95% CI = 95% confidence interval. Only genes which showed statistically significant correlations are depicted

To understand which alterations induced by BT treatment could be linked with patients reported outcomes, changes of gene expression in bronchial biopsies were correlated with changes in the AQLQ and ACQ scores at T2 *versus* T0. A better response to treatment in terms of ΔACQ was associated with lower changes in CD68 (P = 0.0154; r = 0.5651; 95% CI = 0.2160 to 0.7861) and FAP (P = 0.0126; r = 0.4736; 95% CI = 0.1021 to 0.7290) (Fig. 2A). Instead, a higher reduction of PGP9.5 gene expression correlated with a better response to treatment in terms of ΔAQLQ (P = 0.0033; r = − 0.5451; 95% CI = − 0.7712 to − 0.1969) (Fig. 2B). No correlations were observed between changes in the expression of the other genes and changes in AQLQ or ACQ (Additional file 3: Figure S2 and Additional file 4: Figure S3).

### Association between gene expression and clinical parameters 12 months post-BT

In our cohort only 5 patients were classified as non-responders to BT, thus it was not feasible to stratify patients in responders (n = 22) *versus* non-responders (n = 5) to find associations between clinical and molecular data. However, we determined if there were associations between gene expression levels at T0 and T2 and the clinical parameters used to evaluate BT efficacy: number of exacerbations in the 12 months post-BT and ACQ at T12. It was not feasible to consider OCS reduction because only two patients did not reduce OCSs by at least 50%.

**Fig. 3:**
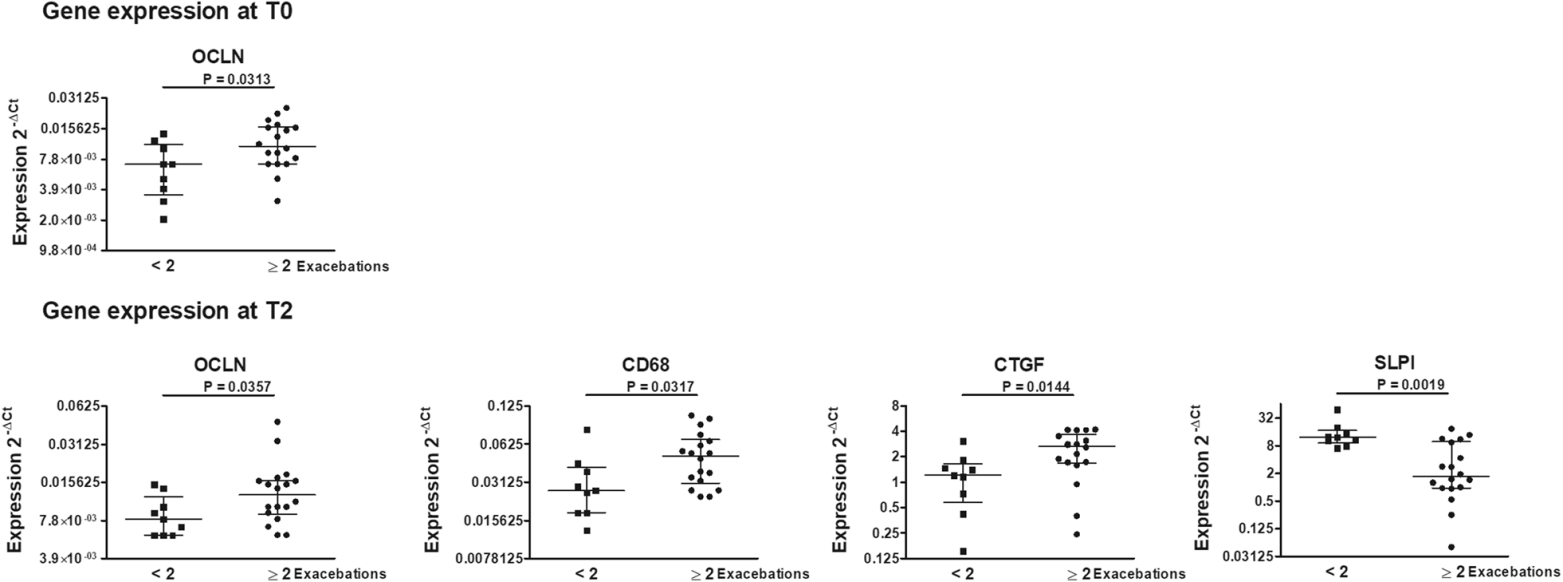
Gene expressions at T0 and T2 associated with the numbers of exacerbations post-BT. Gene expressions in bronchial biopsies at T0 and T2 were calculated by the 2^− ΔCt^ method using the GAPDH as housekeeper gene, classifying patients according to the number of exacerbations during the 12 months of follow up post BT (n = 27). Horizontal lines show the median ± interquartile range (IQR). Data were analyzed by Mann–Whitney U test. Only genes which showed statistically significant associations (p < 0.05) are depicted

Based on the number of exacerbations, statistically significant lower levels of OCLN in bronchial biopsies at T0; lower levels of OCLN, CD68, CTGF and higher levels of SLPI in bronchial biopsies at T2 were observed in patients who had less than 2 exacerbations (p < 0.05, Fig. 3). Additional file 5: Figure S4 and Additional file 6: Figure S5 shows the expression of all the investigated genes at T0 and T2 grouping patients according to the numbers of exacerbations.

Based on ACQ at T12, statistically significant lower levels of COL1A2 in bronchial biopsies at T0 were observed in patients with ACQ < 1.5 (considered as controlled asthma) (Additional file 7: Figure S6). Instead no differences were found analyzing gene expression at T2 (data not shown).

**Fig. 4:**
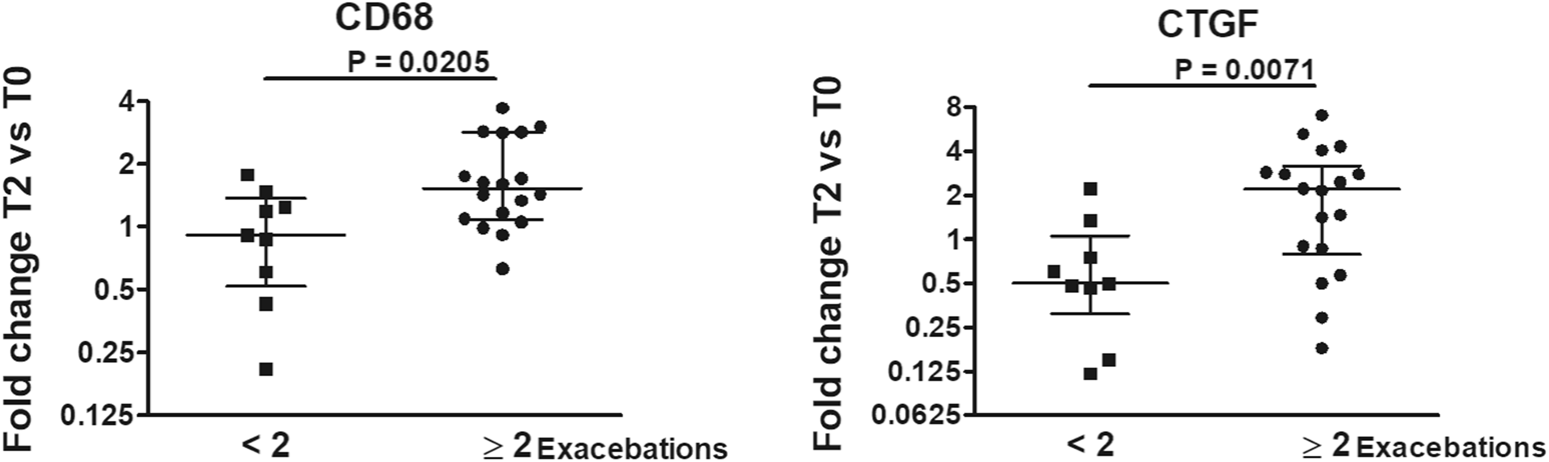
Changes in gene expression at T2 versus T0 associated with the numbers of exacerbations post-BT. Genes whose fold changes in expression in bronchial biopsies at T2 *versus* T0 were different grouping patients according to the numbers of exacerbations experienced during the 12 months of follow up post-BT (n = 27). Horizontal lines show the median ± interquartile range (IQR). Data were analyzed by Mann–Whitney U test. Only genes which showed statistically significant differences (p < 0.05) are depicted

Finally, classification of patients according to the fold changes in gene expression at T2 *versus* T0 revealed that lower changes in CD68 and CTGF mRNAs were observed in patients who had less than 2 exacerbations post-BT (Fig. 4 and Additional file 8: Figure S7 to evaluate all the investigated genes). Instead no differences in the fold changes were found stratifying patients on ACQ values at T12 (data not shown).

## Discussion

This study is divided in two different parts: clinical effects of BT and evaluation of gene expression levels associated to clinical outcomes. Our results confirmed the efficacy of BT in the treatment of severe asthma, as we observed 81% of responders, in line with recent results published by Langton’s group [26].

Notably, our research was conducted in real life and it follows that patients who underwent BT had more severe asthma than patients included in earlier trials in term of rate of exacerbations (6 vs 0.7), AQLQ score (2.5 vs 4.7) and OCS maintenance dose (89% vs 41%) [8, 9].

Regarding clinical outcomes, a significant improvement in asthma symptoms in terms of ACQ scores, AQLQ scores and rate of exacerbations was observed. These results confirm the clinical benefits of BT in line with previous researches [20, 21, 26]. AQLQ improvement at 12 months was statistically significant (p < 0.0001) which is different from what was presented in AIR2 Trial, where AQLQ was statistically borderline and questioned [12, 25, 27–29]. Moreover, we observed a significant reduction in maintenance doses of OCS.

In the scenario of multiple therapeutic approaches for severe asthma treatments, BT still remains the most controversial. One of the biggest objections is the absence of a specific biomarker able to define target population for BT [12]. Currently, BT is used primary in non-eosinophilic phenotype, according to guidelines as there is no usable biological therapy [25]. In our site, BT started in 2012, when the only available biological treatment was omalizumab. Based on clinical criteria for responder definition described in Table 2, we observed 86% of responders in the T2-low cohort and 77% of responders in the T2-high cohort. This finding confirms that BT is a good therapeutic option in T2-low asthma patients. Moreover, it may be effective also in T2-high asthma patients (18% of patients were switched from omalizumab). Thus, our data emerge in contrast with the conception on BT being a treatment only in case of T2-low endotypes or when biological drugs fails [25]. In the real practice, BT represents a therapeutic option for patients with severe asthma despite of asthma endotypes.

The study expands the knowledge regarding the mechanisms of action of BT using gene expression profiling in bronchial biopsy samples. Genes to be investigated were selected through a hypothesis-driven approach based on and expanding data available in the literature [6, 13–17, 20–24].

BT induced a reduction of ACTA2 and an increase of FAP, COL1A1, COL1A2, CD68 mRNAs in the majority of patients suggesting tissue remodelling with a likely decrease in smooth muscle cells and an increase in monocytes/macrophages/dendritic cells and connective tissue. However, these changes seemed to occur irrespective of clinical outcomes, with the exception of the increase in CD68 mRNA levels which were associated with higher numbers of exacerbations post-BT.

### Changes in genes linked to airway remodeling during BT

ACTA2 gene (also known as α-SMA) encodes for smooth muscle actin. Its expression is characteristic of smooth muscle cells and myofibroblasts. There are some reports showing that one of the effects of BT treatment is the reduction of airway α-SMA [6, 13–15, 20, 21, 30, 31]. Herein data confirm this finding at mRNA level in bronchial biopsies from a wide cohort of asthmatic patients after BT treatment.

FAP expression is low in most adult tissues under physiological conditions but it is usually high in reactive stromal fibroblasts. FAP is thought to be involved in the control of fibroblast growth and epithelial-mesenchymal transitions during development, tissue repair, and carcinogenesis. In our knowledge, this is the first report that documented the up-regulation of FAP following BT treatment.

COL1A1 and COL1A2 mRNAs encode pro-alpha I and pro-alpha II chains of type I collagen. Chakir J. and collaborators have reported a reduction in type I collagen thickness below the basement membrane by immunohistochemistry in 9 bronchial biopsies collected from patients at third BT session in comparison to baseline [15]. This alteration persisted after 27 months of follow up [16]. In contrast, we found a huge increase of mRNAs for type I collagen in 89% (COL1A1) and 63% (COL1A2) of bronchial biopsies during BT treatment. Our results at mRNA levels agree with the observations of Pretolani and collaborators showing significantly larger and more intense area stained for collagen in bronchial biopsies at the last BT session *versus* baseline [20]. The explanation for the discordant results could be found in the fact that Chakir J et al. [15]. and Salem J.H. et al. [16]. focused the attention only on the collagen localized under the reticular layer of the basement membrane, while we analyzed the total content of collagen, as well as Pretolani et al. did [20].

Asthma is characterized by airway hyperresponsiveness and it is known that there is a direct correlation with airway remodelling [32]. At cellular level, airway remodelling consists in smooth muscle hyperplasia characterized by a thicker α-SMA layer, epithelial damage and collagen deposition in the subepithelial basement membrane [21, 32–34]. The emerging idea is that the deposition of connective tissue around the airways can determine tissue stiffness, dampening the contractility of α-SMA layer [34, 35]. Based on the present data we speculate that BT treatment could reduce airway hyperresponsiveness decreasing α-SMA layer and increasing fibroblasts with collagen production.

### Changes in genes linked to inflammation during BT

CD68 is mainly expressed by monocytes, macrophages (both M1 and M2 macrophages) and dendritic cells. The increase of CD68 positive cells in bronchial tissues from asthmatic patients compared with healthy controls is known [36]. Moreover, CD68 expression has been previously reported as increased after BT treatment at protein level in bronchial biopsies from 12 asthmatic patients by our group [6], herein confirmed at mRNA level by real-time PCR in a larger cohort of patients.

We did not detect any expression of IL-4, IL-13 and IL-17A mRNAs and a limited expression of IL-5 mRNA in bronchial samples. These cytokines, markers of Th2 and Th17 lymphocytes, have been reported as expressed in samples from asthmatic patients (bronchial biopsies, epithelial brushings, sputum, bronchoalveolar lavage) [2] but usually by means of different techniques: immunohistochemistry and in situ hybridization. One study has investigated the expression of IL-4, IL-5 and IL-13 by PCR: no expression has been detected unless tissue fragments were stimulated ex vivo [45]. Recently, Dr Pretolani and collaborators have reported a decrease in IL-13-positive cells per mm^2^ by immunohistochemistry in bronchial biopsies after BT [31]. Differences in the results can be explained hypothesizing a scarce correlation between mRNA and protein expression and/or a different ability to detect these markers in tissue samples between PCR and immunohistochemistry.

### Changes in genes linked to innervation during BT

PGP9.5 is a protein expressed in the innervation of mucosal glands, smooth muscle and mucosal blood vessels of the lungs [37]. Previous studies, which used PGP9.5 as a marker for the identification and count of nerve C fibers by immunohistochemistry, have reported a reduction of neural innervation after BT treatment [6, 20, 38]. Herein, changes in PGP9.5 mRNA levels during BT treatment were heterogeneous among patients. However, a higher reduction in PGP9.5 mRNA levels correlated with better patients reported outcome in terms of ΔAQLQ, suggesting that a reduction of innervation might be linked with BT efficacy.

### Predictors of outcome 12 months post-BT

To identify predictors of BT efficacy we associated normalized gene expression levels at baseline and T2 as well as changes in gene expression at T2 *versus* baseline with clinical parameters 12 months after BT. At baseline, lower mRNA levels of OCLN and COL1A2 were associated with parameters of better outcome: lower numbers of exacerbations and lower ACQ values post-BT. Moreover, it appeared valuable to quantify gene expression during BT, at the third session of BT. Indeed, lower mRNA levels of OCLN, CD68, CTGF and higher mRNA levels of SLPI at T2, lower changes in CD68 and CTGF mRNA levels at T2 versus T0 resulted associated with fewer exacerbations post-BT. We speculate that the quantification of OCLN, CD68, CTGF and SLPI mRNA could be useful to identify patients at higher risk of exacerbations following BT treatment.

OCLN is a key component of tight junctions, it is involved in the creation of a barrier in airway epithelial cells reducing the transport from apical to basolateral surface. Reduction in OCLN levels has been associated with impaired barrier function in asthma patients [39, 40]. OCLN is also expressed in tight junctions by myelinating Schwann cells protecting peripheral nerves and promoting neurons communication [41]. This is the first study that investigated OCLN in the context of BT treatment and further in-depth analyses will be necessary to elucidate the relationship between tight junction functionality and development of exacerbations.

CTGF is an extracellular matrix protein, involved in tissue regeneration, fibrosis and wound healing; CTGF gene expression has been linked to airway smooth muscle cells [42] and fibroblast to myofibroblast transition [43]. Blocking CTGF has been suggested as a rationale option in patients with asthma [44].

SLPI gene expression has emerged as the most promising marker at baseline to predict response to BT in the work by Ano S et al. [24]. SLPI can inhibit leukocyte elastase, cathepsin G, trypsin and mast cell chymase. Based on Fig. 3 shown in that manuscript [24], it seems even possible to define a cut-off in SLPI expression in bronchial specimens which could distinguish responders from non-responders to BT [24]. We were not able to confirm the usefulness of the quantification of SLPI gene expression at baseline to predict response to BT. However, the quantification of SLPI gene expression levels during BT were the best marker associated with the numbers of exacerbations post-BT, thus confirming a potential value for SLPI.

## Limits and strengths of the study

The limit of the present study is the hypothesis-driven selection of the investigated genes. However, the results are in part consistent with those of previous studies, in part open up new possibilities. A high-throughput approach would allow to discover additional mechanisms of BT treatment. Analysis of the transcriptome has been recently performed on samples from really small cohorts of patients who underwent BT treatment: on cytology brushes from tracheal walls of 5 patients [46], and on bronchial biopsies from 8 patients [24]. Furthermore, Sun Q. et al. used a high-throughput approach on 9 primary airway epithelial cell lines obtained from patients before and after BT treatment and cultured in vitro [47]. In our knowledge this study analyzed the largest cohort of patients who underwent BT, coupling molecular and clinical data at baseline, at the last BT session and after 1 year of follow up. Moreover, the use of real-time PCR allowed us to analyze in each sample a higher number of markers compared with previous studies [6, 13–17, 20, 21, 30, 31].

## Conclusions

In conclusion, our study revealed that BT was effective in 81% of patients irrespective of asthma endotypes and was associated with a reduction in ACTA2 and an increase in FAP, COL1A1, COL1A2 and CD68 gene expression. Quantification of COL1A2, OCLN, CD68, CTGF, SLPI at baseline and at the third BT session might be useful to identify patients at higher risk for exacerbations post-BT. These results are exploratory, further studies are needed to test whether the investigation of the expression of any of these genes could be useful to support clinical practice.

## Supplementary Information

**Additional file 1: Table S1**. List of primers used for real-time PCR. **Table S2**. Gene expression levels in bronchial biopsies at baseline (T0). **Table S3**. Fold changes in gene expression levels in bronchial biopsies between T2 and T0.

**Additional file 2: Figure S1**. Gene expression levels at baseline according to OCS dose. Expressions of the investigated genes in bronchial biopsies at T0 from patients classified in three groups based on the OCS dose: ≤ 5 mg (n = 8); 5 – 25 mg (n = 7); ≥ 25 mg (n = 12). Gene expressions were calculated by the 2− ΔCt method using the GAPDH as housekeeper gene. Horizontal lines show the median ± interquartile range (IQR). Data were analysed by Kruskal-Wallis test. No statistically significant differences were found.

**Additional file 3: Figure S2**. Correlations between fold changes in gene expression and ΔAQLQ during BT treatment. Dot plot visualization of correlations between fold changes in gene expression and differences in AQLQ scores between T2 and T0. Fold changes in gene expression were determined by real-time PCR relative to gene expression at T0 through the 2-ΔΔCt method. Data were analyzed by Spearman's correlation test (n = 27). r = correlation coefficient; 95% CI = 95% confidence interval.

**Additional file 4: Figure S3**. Correlations between fold changes in gene expression and ΔACQ during BT treatment. Dot plot visualization of correlations between fold changes in gene expression and differences in ACQ scores between T2 and T0. Fold changes in gene expression were determined by real-time PCR relative to gene expression at T0 through the 2-ΔΔCt method. Data were analyzed by Spearman's correlation test (n = 27). r = correlation coefficient; 95% CI = 95% confidence interval.

**Additional file 5: Figure S4**. Gene expressions at baseline in patients stratified on exacerbations post-BT. Expression of the investigated genes in bronchial biopsies at T0 from patients classified according to the number of exacerbations during the 12 months after BT (n = 27). Gene expressions were calculated by the 2− ΔCt method using the GAPDH as housekeeper gene. Horizontal lines show the median ± interquartile range (IQR). Data were analyzed by Mann-Whitney U test.

**Additional file 6: Figure S5**. Gene expressions at T2 in patients stratified on exacerbations post-BT. Expression of the investigated genes in bronchial biopsies at T2 from patients classified according to the number of exacerbations during the 12 months after BT (n = 27). Gene expressions were calculated by the 2− ΔCt method using the GAPDH as housekeeper gene. Horizontal lines show the median ± interquartile range (IQR). Data were analyzed by Mann-Whitney U test.

**Additional file 7: Figure S6**. Gene expressions at baseline in patients stratified on the ACQ questionnaire scores post-BT. Expression of the investigated genes in bronchial biopsies at T0 from patients classified on the ACQ questionnaire scores 12 months after BT (n = 27). ACQ < 1.5 controlled asthma; ACQ ≥ 1.5 uncontrolled asthma. Gene expressions were calculated by the 2− ΔCt method using the GAPDH as housekeeper gene. Horizontal lines show the median ± interquartile range (IQR). Data were analyzed by Mann-Whitney U test.

**Additional file 8: Figure S7**. Fold changes in gene expressions in patients stratified on exacerbations post-BT. Fold changes in gene expression (T2 versus T0) in bronchial biopsies grouping patients according to the numbers of exacerbations experienced during the 12 months of follow up post-BT (n = 27). Horizontal lines show the median ± interquartile range (IQR). Data were analyzed by Mann-Whitney U test.

## Data Availability

The data that support the findings of this study are available from the corresponding author upon reasonable request.

## Abbreviations

SRA: Severe refractory asthma
IgE: Immunoglobulin E
BT: Bronchial thermoplasty
AQLQ: Asthma quality of life questionnaire
ACQ: Asthma control questionnaire
TRPV1: Transient receptor potential cation channel subfamily V member 1
TRPV2: Transient receptor potential cation channel subfamily V member 2
UCHL1: Ubiquitin carboxy-terminal hydrolase L1 (also known as PGP9.5)
ACTA2: Alpha smooth muscle actin
CTGF: Connective tissue growth factor
FAP: Fibroblast activation protein alpha
IL-4: Interleukin-4
IL-5: Interleukin-5
IL-6: Interleukin-6
IL-8: IL-13: Interleukin-13
IL-17: Interleukin-17
COL1A1: Alpha-1 type I collagen
COL1A2: Alpha-2 type I collagen
POSTN: Periostin
LGAL3: Galectin-3
OCLN: Occludin
CDH1: Cadherin 1
GAPDH: Glyceraldehyde 3-phosphate dehydrogenase
SLPI: Secretory leukocyte protease inhibitor
OCSs: Oral corticosteroids
