# Supplementary material for: Bronchial thermoplasty in severe asthma: a real-world study on efficacy and gene profiling
Source: Allergy Asthma Clin Immunol. 2022 May 9;18:39. doi: 10.1186/s13223-022-00680-4 (PMC9087992; doi:10.1186/s13223-022-00680-4)
Supplement: Supplementary file 1 — Additional file 1: Table S1. List of primers used for real-time PCR. Table S2. Gene expression levels in bronchial biopsies at baseline (T0). Table S3. Fold changes in gene expression levels in bronchial biopsies between T2 and T0. [file 13223_2022_680_MOESM1_ESM.docx]

**Supplementary Table 1.** List of primers used for real-time PCR.

| **Gene symbol** | **QuantiTect primer assays** |
| --- | --- |
| TRPV1 | QT00046109 |
| TRPV2 | QT00035987 |
| UCHL1/PGP9.5 | QT00092666 |
| ACTA2 | QT00088102 |
| CD45 | QT00028791 |
| CD68 | QT00037184 |
| CTGF | QT00052899 |
| FAP | QT00074963 |
| IL-4 | QT00012565 |
| IL-5 | QT00001435 |
| IL-6 | QT00083720 |
| IL-13 | QT00000511 |
| IL-17A | QT00009233 |
| COL1A1 | QT00037793 |
| COL1A2 | QT00072058 |
| POSTN | QT00023800 |
| LGAL3 | QT01026725 |
| OCLN | QT00081844 |
| CDH1 | QT00080143 |
| SLPI | QT00236117 |
| GAPDH | QT01192646 |

**Supplementary Table 2** Gene expression levels in bronchial biopsies at baseline (T0)

|  | **Expression 2^-ΔCt^** | | |  |  |
| --- | --- | --- | --- | --- | --- |
|  | **Median** | **25% percentile** | **75% percentile** | **Coefficient of variation** | |
| **TRPV1** | 0.013 | 0.008 | 0.022 | 62.5% | |
| **PGP9.5** | 0.007 | 0.004 | 0.013 | 83.9% | |
| **ACTA2** | 0.759 | 0.398 | 1.271 | 90.2% | |
| **CD45** | 0.032 | 0.015 | 0.051 | 61.3% | |
| **CD68** | 0.030 | 0.022 | 0.041 | 42.5% | |
| **CTGF** | 1.317 | 0.881 | 2.780 | 63.9% | |
| **FAP** | 0.037 | 0.001 | 0.006 | 122.0% | |
| **IL-6** | 0.006 | 0.002 | 0.012 | 168.0% | |
| **COL1A1** | 0.026 | 0.013 | 0.039 | 82.3% | |
| **COL1A2** | 0.083 | 0.034 | 0.137 | 104.1% | |
| **POSTN** | 0.025 | 0.014 | 0.049 | 82.9% | |
| **LGAL3** | 0.918 | 0.684 | 1.283 | 42.1% | |
| **CDH1** | 0.354 | 0.258 | 0.398 | 42.5% | |
| **OCLN** | 0.009 | 0.007 | 0.015 | 56.7% | |
| **SLPI** | 14.120 | 9.245 | 20.840 | 66.3% | |

**Supplementary Table 3** Fold changes in gene expression levels in bronchial biopsies between T2 and T0

|  | **Decrease**  **(fold change ≤ 0.5)** | **No change**  **(fold change 0.5 - 2)** | **Increase**  **(fold change ≥ 2)** |
| --- | --- | --- | --- |
| **TRPV1** | 5/27 | 17/27 | 5/27 |
| **PGP9.5** | 8/27 | 10/27 | 9/27 |
| **ACTA2** | 14/27 | 9/27 | 4/27 |
| **CD45** | 5/27 | 16/27 | 6/27 |
| **CD68** | 2/27 | 20/27 | 5/27 |
| **CTGF** | 8/27 | 8/27 | 11/27 |
| **FAP** | 0/27 | 8/27 | 19/27 |
| **IL-6** | 10/27 | 7/27 | 10/27 |
| **COL1A1** | 1/27 | 2/27 | 24/27 |
| **COL1A2** | 1/27 | 9/27 | 17/27 |
| **POSTN** | 4/27 | 17/27 | 6/27 |
| **LGAL3** | 3/27 | 21/27 | 3/27 |
| **CDH1** | 1/27 | 22/27 | 4/27 |
| **OCLN** | 3/27 | 17/27 | 7/27 |
| **SLPI** | 8/27 | 13/27 | 6/27 |
