# Supplementary figures and images for: Bronchial thermoplasty in severe asthma: a real-world study on efficacy and gene profiling
Source: Allergy Asthma Clin Immunol. 2022 May 9;18:39. doi: 10.1186/s13223-022-00680-4 (PMC9087992; doi:10.1186/s13223-022-00680-4)

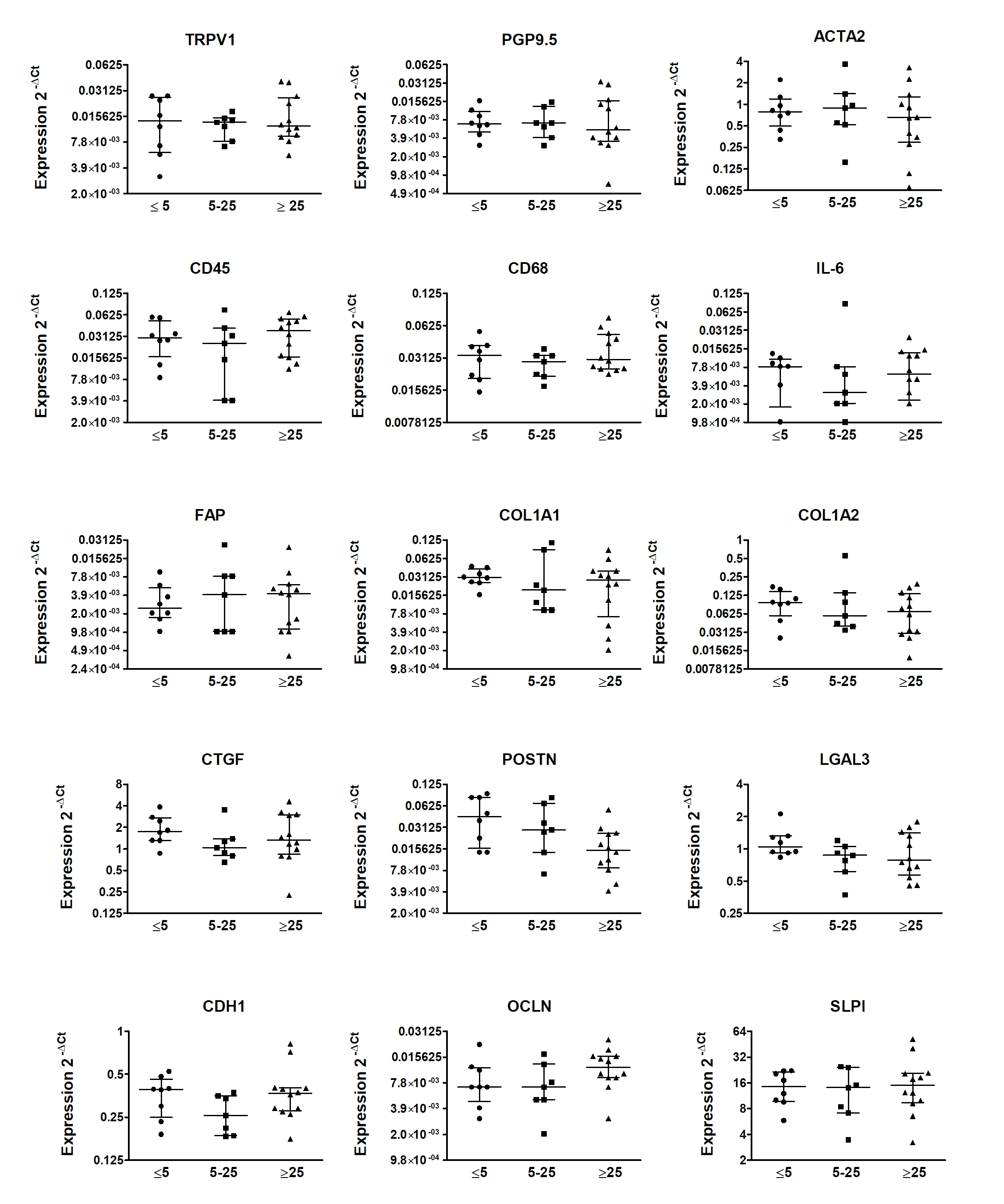

Supplement: Supplementary file 2 — Additional file 2: Figure S1. Gene expression levels at baseline according to OCS dose. Expressions of the investigated genes in bronchial biopsies at T0 from patients classified in three groups based on the OCS dose: ≤ 5 mg (n = 8); 5 – 25 mg (n = 7); ≥ 25 mg (n = 12). Gene expressions were calculated by the 2− ΔCt method using the GAPDH as housekeeper gene. Horizontal lines show the median ± interquartile range (IQR). Data were analysed by Kruskal-Wallis test. No statistically significant differences were found. [file 13223_2022_680_MOESM2_ESM.tif]

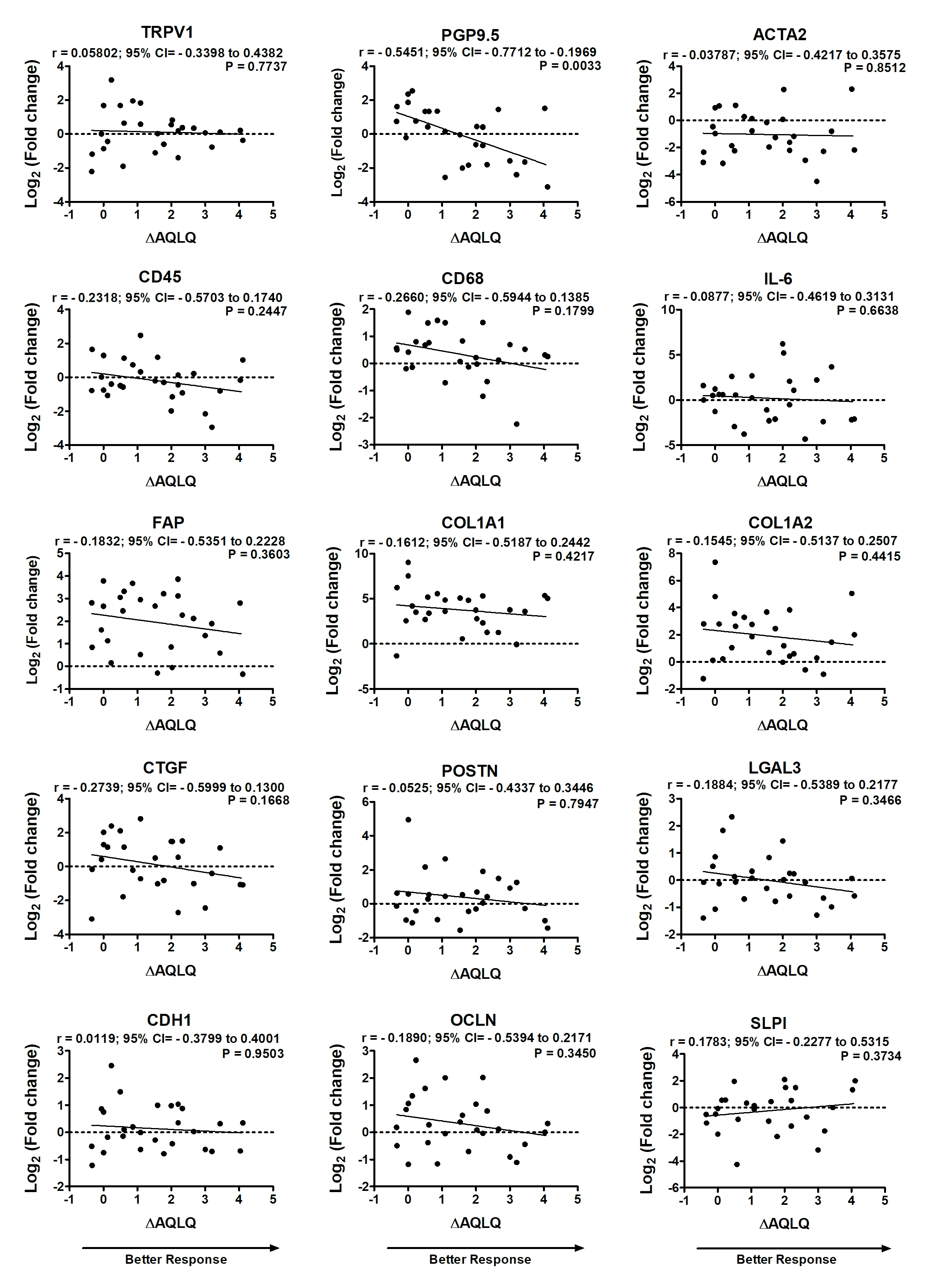

Supplement: Supplementary file 3 — Additional file 3: Figure S2. Correlations between fold changes in gene expression and ΔAQLQ during BT treatment. Dot plot visualization of correlations between fold changes in gene expression and differences in AQLQ scores between T2 and T0. Fold changes in gene expression were determined by real-time PCR relative to gene expression at T0 through the 2-ΔΔCt method. Data were analyzed by Spearman's correlation test (n = 27). r = correlation coefficient; 95% CI = 95% confidence interval. [file 13223_2022_680_MOESM3_ESM.tif]

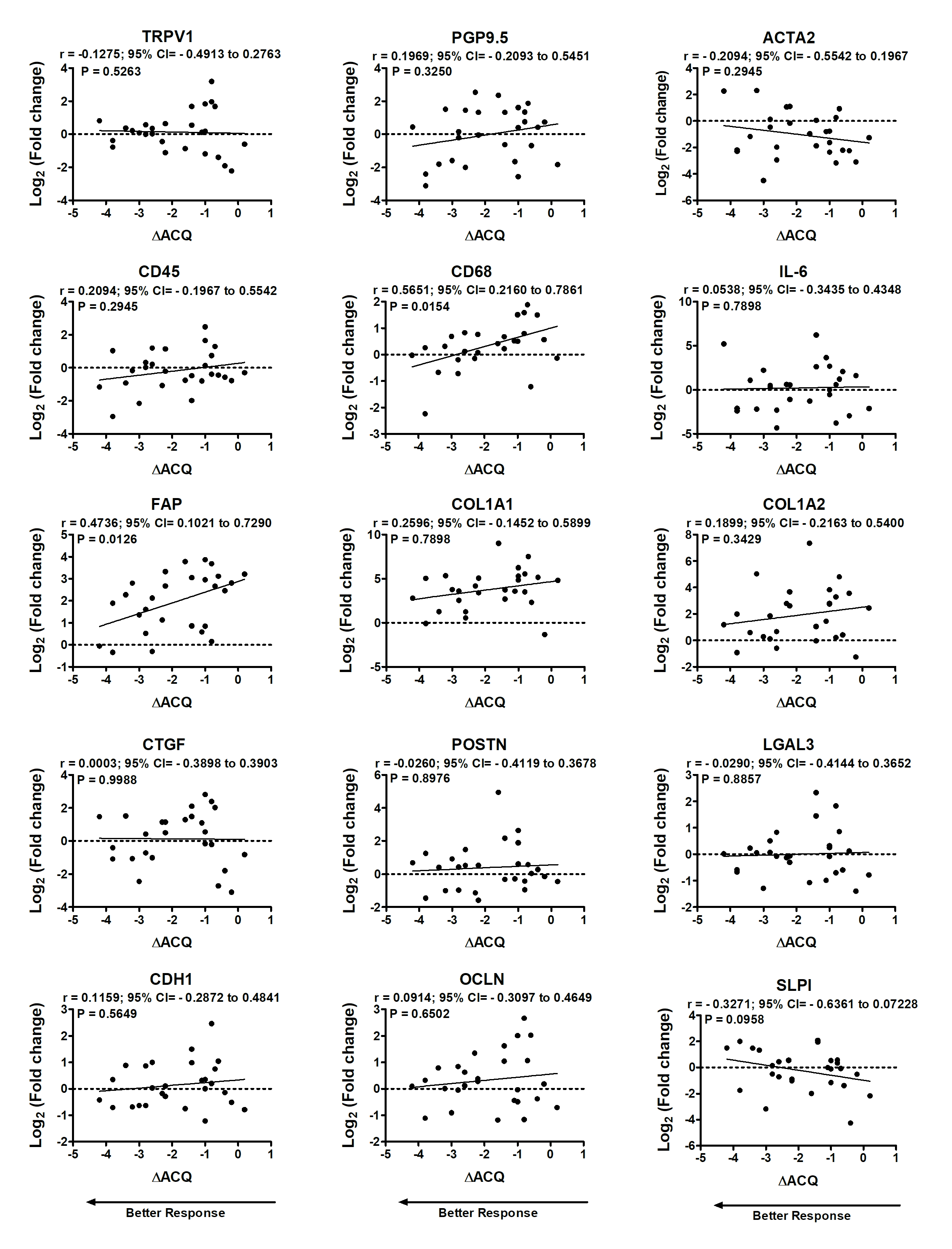

Supplement: Supplementary file 4 — Additional file 4: Figure S3. Correlations between fold changes in gene expression and ΔACQ during BT treatment. Dot plot visualization of correlations between fold changes in gene expression and differences in ACQ scores between T2 and T0. Fold changes in gene expression were determined by real-time PCR relative to gene expression at T0 through the 2-ΔΔCt method. Data were analyzed by Spearman's correlation test (n = 27). r = correlation coefficient; 95% CI = 95% confidence interval. [file 13223_2022_680_MOESM4_ESM.tif]

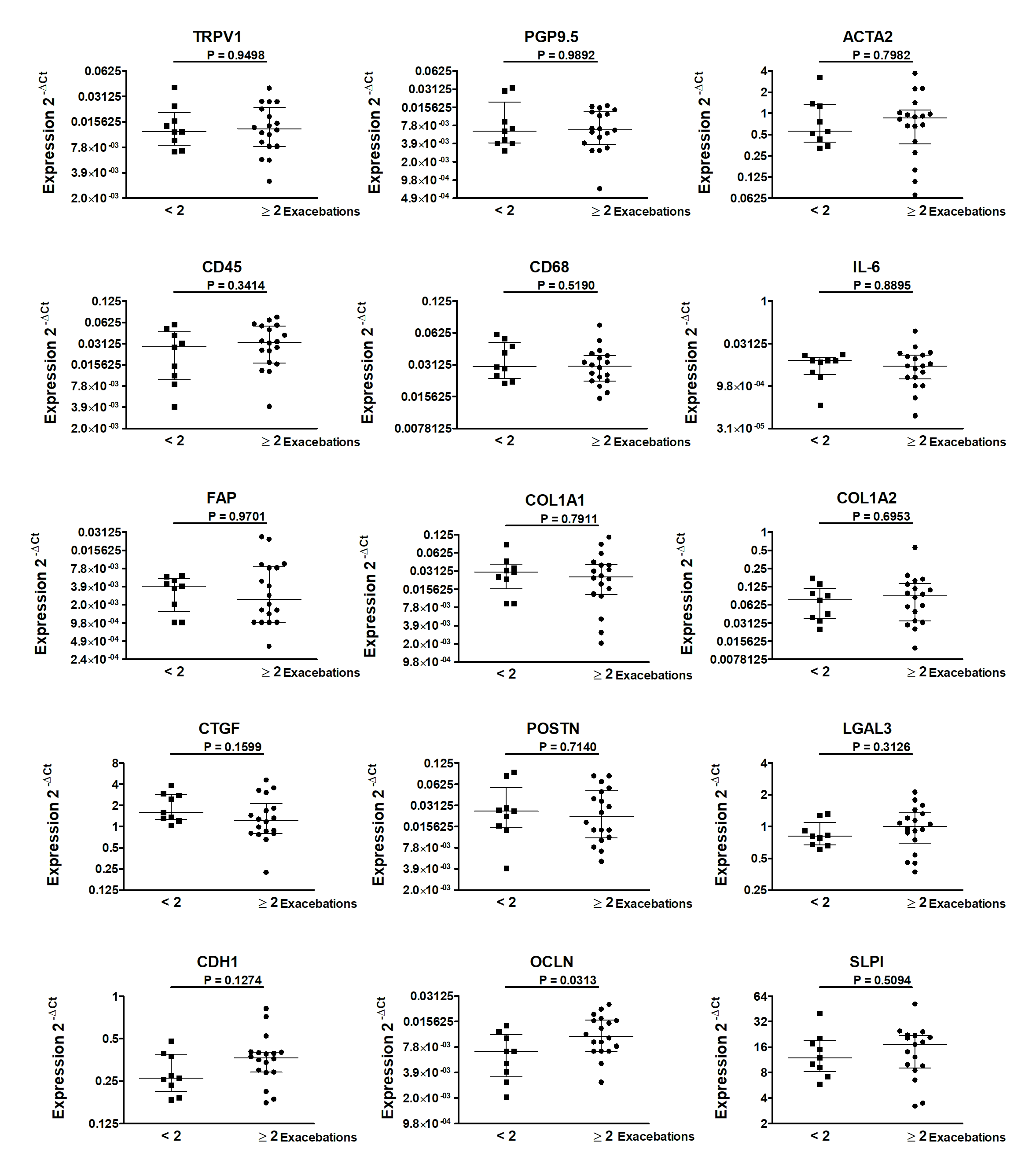

Supplement: Supplementary file 5 — Additional file 5: Figure S4. Gene expressions at baseline in patients stratified on exacerbations post-BT. Expression of the investigated genes in bronchial biopsies at T0 from patients classified according to the number of exacerbations during the 12 months after BT (n = 27). Gene expressions were calculated by the 2− ΔCt method using the GAPDH as housekeeper gene. Horizontal lines show the median ± interquartile range (IQR). Data were analyzed by Mann-Whitney U test. [file 13223_2022_680_MOESM5_ESM.tif]

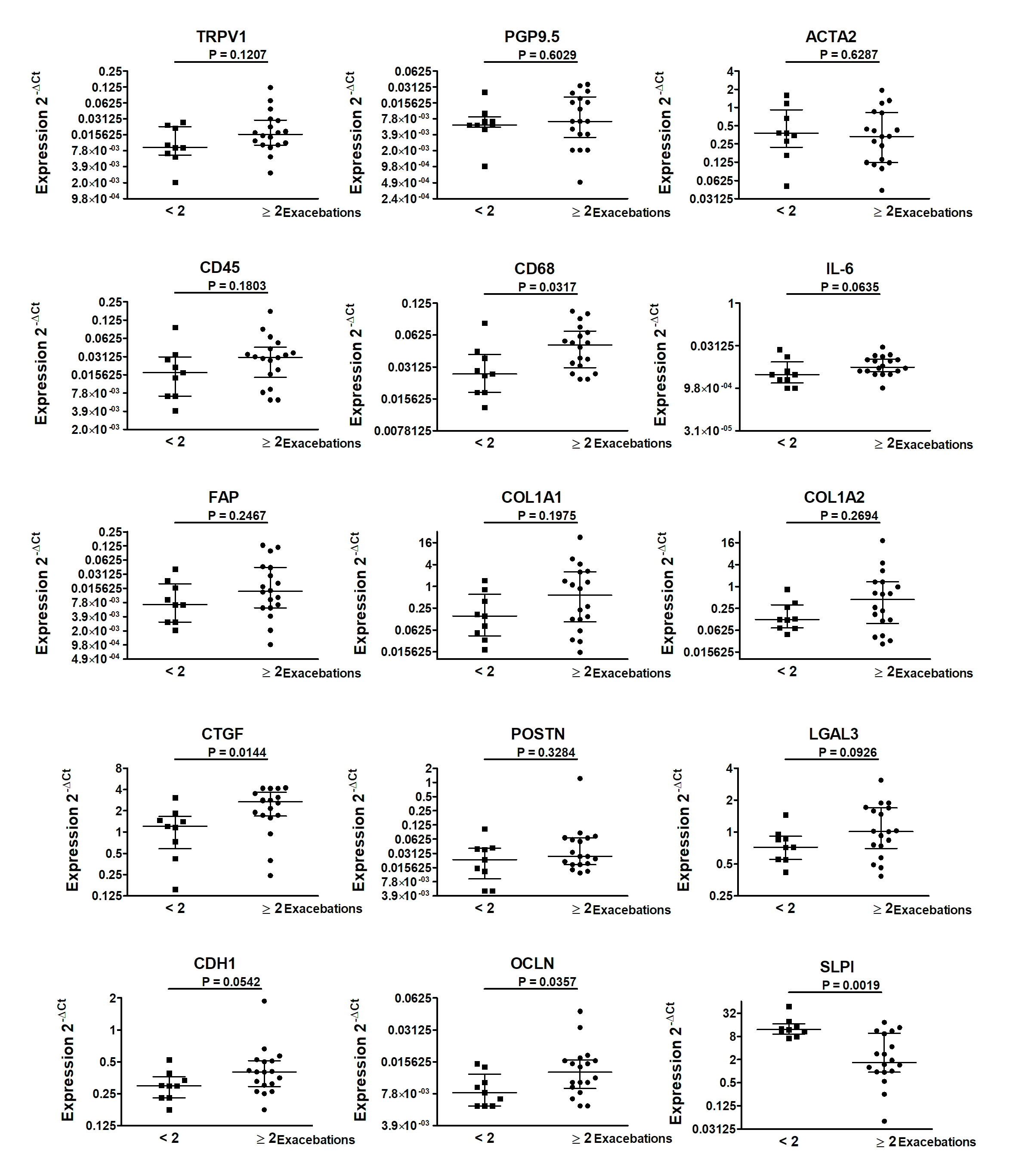

Supplement: Supplementary file 6 — Additional file 6: Figure S5. Gene expressions at T2 in patients stratified on exacerbations post-BT. Expression of the investigated genes in bronchial biopsies at T2 from patients classified according to the number of exacerbations during the 12 months after BT (n = 27). Gene expressions were calculated by the 2− ΔCt method using the GAPDH as housekeeper gene. Horizontal lines show the median ± interquartile range (IQR). Data were analyzed by Mann-Whitney U test. [file 13223_2022_680_MOESM6_ESM.tif]

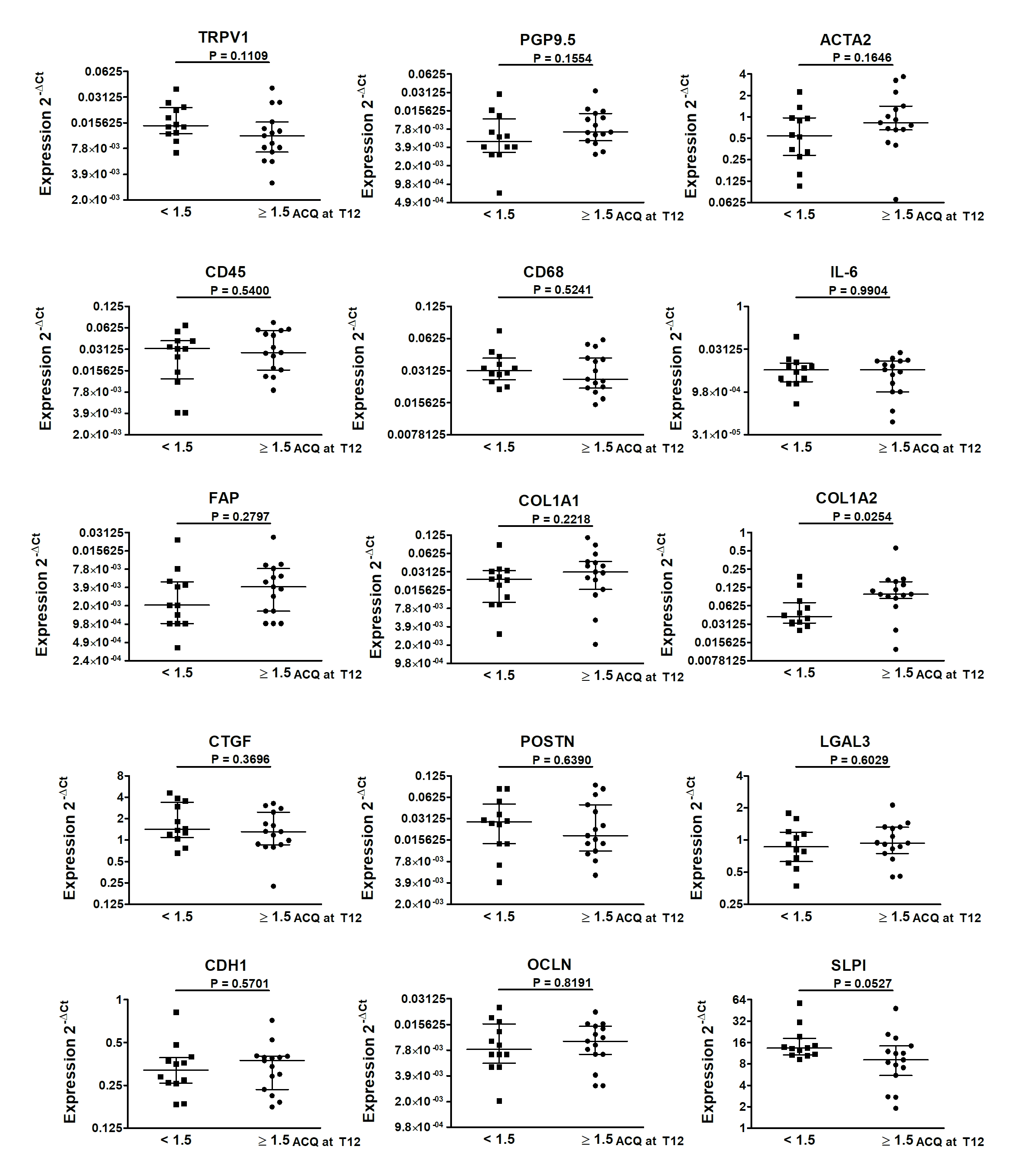

Supplement: Supplementary file 7 — Additional file 7: Figure S6. Gene expressions at baseline in patients stratified on the ACQ questionnaire scores post-BT. Expression of the investigated genes in bronchial biopsies at T0 from patients classified on the ACQ questionnaire scores 12 months after BT (n = 27). ACQ < 1.5 controlled asthma; ACQ ≥ 1.5 uncontrolled asthma. Gene expressions were calculated by the 2− ΔCt method using the GAPDH as housekeeper gene. Horizontal lines show the median ± interquartile range (IQR). Data were analyzed by Mann-Whitney U test. [file 13223_2022_680_MOESM7_ESM.tif]

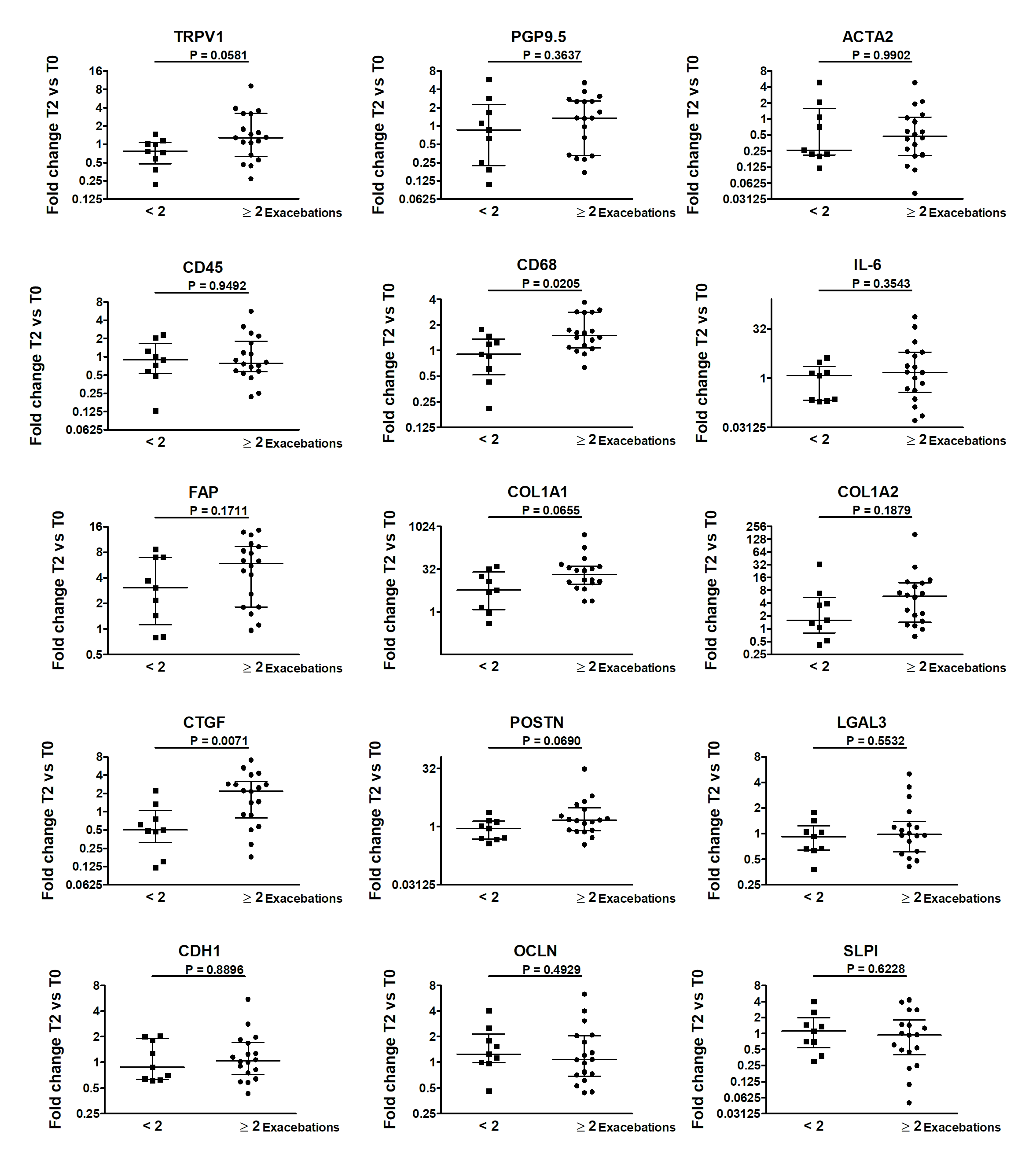

Supplement: Supplementary file 8 — Additional file 8: Figure S7. Fold changes in gene expressions in patients stratified on exacerbations post-BT. Fold changes in gene expression (T2 versus T0) in bronchial biopsies grouping patients according to the numbers of exacerbations experienced during the 12 months of follow up post-BT (n = 27). Horizontal lines show the median ± interquartile range (IQR). Data were analyzed by Mann-Whitney U test. [file 13223_2022_680_MOESM8_ESM.tif]
